# Internet-based psychoeducation for bipolar disorder: a qualitative analysis of feasibility, acceptability and impact

**DOI:** 10.1186/1471-244X-12-139

**Published:** 2012-09-13

**Authors:** Ria Poole, Sharon A Simpson, Daniel J Smith

**Affiliations:** 1 Institute of Psychological Medicine and Clinical Neurosciences, Cardiff University School of Medicine, Monmouth House, University Hospital of Wales, Heath Park, Cardiff, CF14 4DW, UK; 2 South East Wales Trials Unit, Institute of Translation, Innovation, Methodology and Engagement, Cardiff University School of Medicine, 7th floor Neuadd Meirionnydd, Heath Park, Cardiff, CF14 4YS, UK; 3 Institute of Health and Wellbeing, University of Glasgow, Academic Unit of Mental Health and Wellbeing, Gartnavel Royal Hospital, 1055 Great Western Road, Glasgow, G12 0XH, UK

**Keywords:** Bipolar disorder, Patient education, Internet, Feasibility, Patient experiences, Qualitative

## Abstract

**Background:**

In a recent exploratory randomised trial we found that a novel, internet-based psychoeducation programme for bipolar disorder (*Beating Bipolar*) was relatively easy to deliver and had a modest effect on psychological quality of life. We sought to explore the experiences of participants with respect to feasibility, acceptability and impact of *Beating Bipolar.*

**Methods:**

Participants were invited to take part in a semi-structured interview. Thematic analysis techniques were employed; to explore and describe participants’ experiences, the data were analysed for emerging themes which were identified and coded.

**Results:**

The programme was feasible to deliver and acceptable to participants where they felt comfortable using a computer. It was found to impact upon insight into illness, health behaviour, personal routines and positive attitudes towards medication. Many participants regarded the programme as likely to be most beneficial for those recently diagnosed.

**Conclusions:**

An online psychoeducation package for bipolar disorder, such as *Beating Bipolar*, is feasible and acceptable to patients, has a positive impact on self-management behaviours and may be particularly suited to early intervention. Alternative (non-internet) formats should also be made available to patients.

## Background

There is considerable potential for information technology within healthcare (such as e-Health interventions) to improve the capacity of patients to self-manage long-term conditions [1]. Over 30 million people in the UK now access the internet every day [2] and policy-makers are eager to harness the power of the internet to allow individuals to take a more active role in their own care [3,4]. One potential disadvantage of face-to-face group psychoeducation is the cost of therapist time, training and travel. In collaboration with patients with bipolar disorder, their families and health professionals we have developed an internet-based psychoeducational intervention called “Beating Bipolar” [5]. This work has built on the success of group psychoeducation interventions for bipolar disorder (focusing on illness awareness, adherence to treatment, early detection of recurrence and lifestyle regularity), which have emerged as an effective treatment option for long-term management [6-9].

There have been recent studies which have evaluated group-based psychoeducation for bipolar disorder, such as the randomised controlled trial by Castle et al., 2010 [10], which found that the treatment group had a significantly reduced rate of relapse, and the randomised controlled trial by Eker and Harkin [11] which found that patients’ adherence to medication had significantly increased after group psychoeducation. Larger randomised controlled trials of group psychoeducation for bipolar disorder found that manic symptoms may be reduced for up to 2 years following the intervention [12], and a study by Bauer et al., 2006 [13,14], found that at 3 years follow-up the treatment group had significantly fewer weeks in a bipolar episode, significantly improved social functioning and significantly improved mental quality of life.

*Beating Bipolar* involves a blending of different delivery mechanisms, with initial face-to-face delivery, followed by internet-based interactive delivery of factual content and ongoing support via a web forum [15]. The key areas covered are: i) the accurate diagnosis of bipolar disorder; ii) the causes of bipolar disorder; iii) the role of medication; iv) the role of lifestyle changes; v) relapse prevention and early intervention; vi) psychological approaches; vii) gender-specific considerations; and, viii) advice for family and carers. In the clinical trial [16] participants were given access to each of the modules in turn every 2 weeks and were encouraged to discuss the content of each module within the discussion forum. The programme content is similar in focus to Bauer and McBride’s Life Goals Program [17] and Colom and Vieta’s group psychoeducation intervention for bipolar disorder [18]. In a recent exploratory randomised trial we found that *Beating Bipolar* was relatively easy to deliver, engaging and had a modest effect on psychological quality of life [16]. Here we report a qualitative study which aims to explore more fully issues relating to the feasibility, acceptability and impact of the intervention.

## Methods

### Design

These qualitative interviews were carried out for the evaluation of the phase II randomised controlled ‘BIPED’ (Bipolar Interactive PsychoEDucation) trial, Current Controlled Trials registration number ISRCTN81375447, approved by the South East Wales NHS Research Ethics Committee [15].

The qualitative methodology employed for this research was consistent with a pragmatic approach, with a view to ensuring a suitable fit between research methods and the research questions posed [19]. Given that we were assessing the feasibility, acceptability and impact of the intervention from participants’ perspectives, we decided that semi-structured interviews conducted in a flexible and responsive manner were the most appropriate method of data collection and thematic analysis would be most appropriate for analysing data. We sought to understand people’s own individual experiences of interacting with the programme as well as to address questions relating to the feasibility and acceptability of the intervention, consistent with key objectives within process evaluation literature [20].

In our results section we report numbers of participants whose responses generated themes, only where it enhances the data by highlighting the commonality of themes among participants or deviant cases. The results presented here are a synthesis of the themes identified within the domains of feasibility, acceptability and impact.

### Recruitment

We interviewed participants who were in the intervention arm of the trial until saturation of themes was achieved during concurrent analysis. We sought feedback from participants who completed all or most of the programme and also from those who chose not to complete the programme. Participants were approached initially by letter, followed by a telephone call to arrange a suitable time for the telephone interview. Prior to selecting participants for interview we collected computer-generated programme usage information. We considered those participants who completed more than half the programme to be “high users”, and those participants who completed less than half the programme to be “low users”. This was so that we could identify participants’ level of engagement with the programme prior to interview, to sensitively enquire about barriers or facilitators to engagement.

### Data collection

Semi-structured interviews covered a number of key areas (see Appendix 1): the implementation and receipt of the intervention; the acceptability and perceived usefulness of various components of the intervention; the impact of the programme; and recommendations for improvement. The interview schedule was used as a guide, but interviews were conducted flexibly to enable participants to explore topics freely. All interviews were recorded.

### Analysis

Data were transcribed verbatim and transcripts were coded and analysed. We employed thematic analysis techniques where transcripts were closely examined to identify themes and categories [19,21,22]. Our pragmatic approach to thematic analysis has been informed by the method as it is described by Braun and Clarke, 2006 [22]. Employing a semi-structured interview schedule provided a focus for the interviews and the themes which consequently emerged were relevant to our research questions. We identified themes as being salient responses which relate to our research questions and may also occur as patterned responses within the data. The coding framework developed in a responsive manner to the themes elicited within each interview and was systematically reviewed and refined as it was applied to the data. Patterns within and across themes were explored throughout the analytic process.

The main coding categories to some extent reflected the questions asked during the interviews as well as emerging trends in the data evident from the prevalence of certain categories and the reiteration of particular points of view. Agreement on concepts was sought between members of the research team to ensure reliability, and the interviews and coding framework were scrutinised until no new insights emerged from the data. RP, who has a background in psychology, conducted the interviews and led the analysis. SS, who has a background in psychology, and DS, who has a background in psychiatry, each read four different manuscripts. The coding framework was discussed throughout its development within fortnightly meetings of the three researchers to ensure that concepts were appropriately identified and described. There were no notable disagreements between researchers regarding the identification and description of concepts within the analysis.

The interviewing was iterative; where new themes emerged we incorporated them into the interviews. Interviews continued until all the themes were saturated. Analysis was supported by the use of the qualitative analysis computer software NVivo version 8 [23].

## Results

Twenty of 24 participants from the intervention arm of the trial took part in the interviews. Fourteen were high users of the programme (13 completed all 8 modules; 1 participant completed modules 1–6) and 6 were low users of the programme (1 participant completed 3 or 4 modules; 2 participants completed the first 2 modules; 3 participants only attempted the first module). Of the high users 8 were male and 6 were female, and of the low users 5 were female and 1 was male. Participants’ age range was between 20 and 65 years (See Table 1).

**Table 1:** Characteristics of interview participants

	**High users**	**Low users**
**Age range**	20-65 years	20-65 years
**Male**	8	5
**Female**	6	1
**Total**	14	6

There were several key themes and sub-themes which emerged from the data. In this paper we focus on the main themes relating to the domains of feasibility, acceptability and impact.

### Feasibility

#### Accessibility and flexibility

Computer literate participants who had access to a private computer and were well enough to engage with the programme found the programme feasible to undertake and complete. Many participants valued the programme’s ease of use and access, and commented that it ran smoothly online. Participants specifically liked being able to access the programme in their own time, at their own pace, and having the option to revisit modules. Some commented that they appreciated having the option to share content by inviting others to look at the programme.

“You can share it and invite other people to sort of look at bits of it with you as well, you couldn’t really invite someone along to a group meeting, could you […] I felt able to engage with it when it was just me and the computer… because in a way I’m very familiar with engaging with the computer.”

PID71, female, high user

Eighteen participants stated that they regarded themselves to be competent in using a computer. Two participants (1 low and 1 high user) reported not being sufficiently computer literate to engage fully with the programme; the high user completed all the modules, but couldn’t access the forum because she regarded it to be too technical for her. Only 5 participants reported difficulties with accessing the programme because of either a reluctance to use a computer or issues surrounding arrangements to access a computer.

#### The effect of illness on engagement with the programme

The mood of some participants at the time of undertaking the programme impacted negatively on their engagement with it. For some, low mood was a motivation to engage more fully with the programme because of a desire to find meanings and solutions for their depressive symptoms. Others reported that low mood compromised their concentration and ability to engage fully, either because confronting the illness made them feel low or they feared experiencing an episode of the illness through learning about bipolar disorder when well.

“I got depressed when I was doing it because, like, it brings it home that you’re ill, cos you can forget about it, you know. […] and I got the same symptoms as people who was on there […] it just brings it home to you then, you know, and you tend to forget about it in real life and you just hide away when you’re ill and come out smiling and happy when you’re okay.”

PID47, male, high user

Of the 7 participants interviewed who did not complete all the modules (6 low users and 1 high user) 4 participants reported experiencing difficulty with concentrating on the programme. Three participants reported becoming less well during the programme, and became distracted and lost motivation to complete it.

#### The importance of accessing the programme in a private environment

The majority of participants accessed the programme from their homes and found this to be acceptable; however, several participants noted that accessing the programme in a private environment was important. Five participants accessed the programme in a public venue, such as a library, hospital, internet café or university. Two participants (low users) who used a public computer felt that their privacy was compromised. Four participants specifically appreciated the privacy and anonymity of the online programme.

### Acceptability

#### Clarity and quality of content

Many participants commented that the programme’s interface was professional and clear. Many felt that the information presented within the modules was relatively easy to follow, comprehensive and of good quality. Participants found the pace of the modules acceptable, and most felt that the gap of 2 weeks between modules was appropriate. A few participants reported feeling impatient to receive the next module at times, but expressed their appreciation that the time between modules enabled them to engage with new concepts and knowledge.

#### “What did you like about the programme?”

“Everything. Um, I enjoyed the clarity of the content and the way there was a lot of […] information available at many levels […] at every level of possible understanding, and it was very up to date as well.”

PID63, male, high user

#### Dislike of actors’ acting

Although some participants reported that they appreciated the videos of the “talking heads”, one theme concerned the appropriateness of using actors and the quality of the acting within these video clips. Many participants felt that these clips were scripted, rather than talking from personal experience, and would have preferred either more convincing and naturalistic acting or people with bipolar disorder speaking from their own experiences.

“I didn’t like the staged-ness […] you could tell they’d done it so many times they were probably on take 500 because someone had forgotten their lines, and it lost a little bit of its authenticity, […] and I think perhaps it might be better to get the actors out of there and get the real ones in there because we felt we could spot them, as people who have got it.”

PID50, female, high user

#### Difficulty with the interactive “life chart” exercise

Another theme emerged with respect to one of the interactive exercises within the programme during which participants were invited to complete an online “life chart” documenting their pattern of relapse. Seven participants criticised it as being too restrictive and difficult to complete, for example, when their pattern of illness was predominantly mixed affective or where they had experienced a large number of relapses. Some participants also found it difficult to remember when past episodes had occurred. One participant found it emotionally difficult to remember past episodes, and was reluctant to recall her difficult experiences because she was scared that the act of remembering may trigger a depressive episode.

“I can remember a timeline […] that did kerfuffle me a bit, remembering back all the bad stuff, wasn’t good. […] I’ve done some stupid stuff, overdoses and stuff, and I’ve got a little girl now I can’t be thinking about stuff like that. And I can’t afford to be, I mean my best mate died in January and I can’t grieve over her cos I’m too scared of sinking in that hole again […].”

PID33, female, low user

#### Lack of activity on the forum

Many participants described the forum as being too quiet and lacking the critical mass for worthwhile conversations or an incentive to log in to it regularly. Some participants expressed feeling self-conscious and lacking the confidence to communicate with others within the context of the forum, and hence were reluctant to do so. Others expressed their uncertainty of the purpose of the forum, and felt that it would benefit from more input from medical professionals.

“I think initially there was only 2 of us putting things back and forth and I think once we realized we were the only 2 we quickly retreated as well. […] I found it really quiet to be honest, that’s the best way to describe it […] if there could be some external, you know, perhaps somebody running the programme to kick the topics off, as opposed to just sort of saying “please discuss”, ask proper questions […] get somebody who’s in charge there or involved in the project to be specific to get the conversations starting.”

PID50, female, high user

#### The presentation of lithium within the medication module

Some participants reported a strong dislike of the presentation of Lithium within the medication module. They felt that Lithium was presented too often without discussion of the serious problems relating to Lithium use, and that it shouldn’t be presented as the drug of choice for bipolar disorder. Many participants felt that other drugs were either not discussed or not discussed enough. Participants suggested that instead of highlighting lithium as a main drug the module should present a more in-depth drug review.

“The one criticism I would have is that they were pushing lithium rather too much. […] I thought well maybe that’s a little bit biased, you know, that there are a lot less side effects with some, so I thought maybe it was some sort of um pharmaceutical company that was involved with that […] if you could sort of try and do perhaps a bit of a drug review with the side effects that people are likely to suffer from […] it was almost like it was a lithium show sort of thing.”

PID44, female, high user

#### Preferences for alternatives to the computer-based format

Although overall most participants found the programme acceptable, some commented that they would have preferred an alternative to the computer-based format as they were resistant to using a computer. Two participants commented that because they belong to an older generation they preferred face-to-face communication over online communication.

“I suppose I just like more face-to-face stuff, […] I mean I’m 63, it’s the younger generation that’s much more accepting of this technology and they use it for everything, but I think I just prefer more face-to-face stuff.”

PID53, female, high user

Some participants would have preferred to have read the information and others would have preferred the social interaction of a face-to-face psychoeducation group.

“I didn’t like the fact that I had to watch, watch and listen, um, you know it’s almost like watching a TV programme, you know, I’d have to watch a presentation or people talking. I much prefer to read information. […] I watch very little television, I mean 15 or 20 minutes my attention span’s filled and that’s about it.”

PID61, male, low user

All participants were asked whether they would prefer internet-based or group-based face-to-face psychoeducation for bipolar disorder (where there may be up to 15 people with bipolar disorder learning together under the direction of a clinician). Of those who stated a preference, 8 said that they would prefer *Beating Bipolar* and 8 preferred a group-based intervention.

#### Internet-based psychoeducation lacks the sociability of group-based learning

Many stated a preference for the sociability of group-based learning, and commented that they would be more stimulated by learning with and from others through group work and face-to-face discussions than by learning on their own. Some suggested that the opportunity to exchange experiences of bipolar disorder within a group may provide social support, an opportunity to make friends and learn from others’ experiences, and may reduce any feelings of isolation.

“Personally I’d be more sort of geared towards learning with others and learning from others. […] it’s just because I don’t ever talk about it in my day to day life with anyone so it’s nice to be able to have people you can openly talk about it to.”

PID7, female, low user

#### Groups of people with mental illness as socially unappealing

All 8 participants who stated a preference for online as opposed to group-based psychoeducation reported that group meetings for people with mental illness were unappealing, and that they would not find support group meetings to be useful. Five participants reported that they did not see themselves as mentally ill, or did not identify with others with mental illness and held the view that others with mental illness are more “ill” than they were.

“I don’t like groups of people, and groups of people who are mentally ill just don’t appeal to me at all. … I don’t go to support groups, I don’t find those sorts of things useful, reminds me too much of hospital.”

PID24, female, low user

Some participants considered that attending a group meeting with people with bipolar disorder would be depressing and frightening.

“I don’t like the idea of sitting in a room with manic depressives, I just don’t like the room, I don’t like the thought of it. It’s just so miserable. A room full of people like me … no.”

PID33, female, low user

Two participants who related their previous experiences of attending group meetings with others who had bipolar disorder remarked that seeing others who were more ill than they were reminded them of how unwell they could become, and were frightened to think that they may deteriorate to the level of those who appeared to be heavily medicated or looked very unwell.

“I can’t say everybody’s the same, my own opinion, the thought of going into a room with 15 people who’ve got bipolar would frighten the life out of me […] It frightens you. It frightens you to think you might deteriorate to that level, you know. I just thank God, cross my heart, that I have not dropped so low that I could be hospitalized or anything, but I’ve seen people who have been hospitalized and it’s not a nice sight […] The heavily medicated, they look like zombies, you know, and I just thank God it hasn’t happened to me yet.”

PID47, male, high user

#### Internet-based psychoeducation may be more acceptable than group-based psychoeducation for those recently diagnosed

Some participants suggested that online psychoeducation would be more acceptable than group-based psychoeducation for those who were newly diagnosed with bipolar disorder. In addition to the perception that meeting with a group of people with mental illness may not appeal to those in the early stage of their illness, online psychoeducation can provide anonymity and an opportunity to take a break from the programme if they felt uncomfortable or lacked concentration.

“In the beginning I would have preferred to gone online. That is because from doing an online programme I would realize that they don’t all sit there in straitjackets, um, I would realize that they’re normal people. […] in the beginning if anyone had said you’re going to go to sit in a group with a load of other people with bipolar I would have gone “not on your nelly”. The anonymity of the online thing is absolutely perfect […] Frightened to death […] if I saw, I just mentioned 2 people there, had they been there on my first meeting I would not have gone back again. I would have been too frightened […] Now I’d be happy to go to a group but not newly diagnosed.”

PID50, female, high user

### Impact

#### Minimal contribution to existing knowledge for those with a long-standing diagnosis

The majority of participants reported benefitting from the programme. Some commented that the programme reinforced or consolidated their existing knowledge of bipolar disorder, although almost all participants were not newly diagnosed with bipolar disorder and many felt that the programme contributed minimally to their understanding.

#### Potential greater impact for those with a new or very recent diagnosis

Many participants felt that the programme would be particularly useful for those who were more recently diagnosed. Some expressed that they would have appreciated the programme in the early stages of their illness as they didn’t have sufficient information on bipolar disorder available to them at that time.

“I think it would be most useful for someone who was newly diagnosed, but for somebody like me it wasn’t really teaching me anything I didn’t already know.”

PID24, female, low user

#### Greater knowledge of bipolar disorder

Even though the programme contributed minimally to most participants’ understanding of bipolar disorder, many participants reported that they had learned something new as a result of the programme. As a result of the lifestyle module some participants recognised what may trigger an episode of bipolar disorder, such as stress, alcohol, and lack of sleep or moderate exercise. Two participants remarked that the programme (particularly the introductory module) had contributed to a greater acceptance of the illness.

“I think maybe it impacts perhaps indirectly in so much as it has facilitated, although I can still feel desperate at times, […] I accept it far more perhaps than I used to, I realize that it’s not being, you know, a complete and utter shit basically, it is actually because I’ve got a mood swing and you know or things aren’t as stable as they ought to be and that you know it will pass, which is again part of the learning curve I guess.”

PID52, male, high user

#### Improved self-awareness

Many participants expressed the view that the programme encouraged them to think about self management techniques, how to monitor their thoughts and feelings, and how to regulate their behaviour.

“I feel now that I would be more aware of the changes in me, but that’s only a feeling because of course I haven’t had another episode to actually put that to the test. […] So I do feel in that sense […] it’s been a good experience to do this, to actually recognize when my thought processes, you know, might be going off track.”

PID53, female, high user

#### Behaviour change as a result of the programme

Some participants adapted their health behaviour, lifestyle or routine as a result of the programme; specifically because of the modules on lifestyle changes and relapse prevention. Since undertaking the programme, some participants reported implementing the following changes: creating and maintaining a regular routine, quitting smoking, reducing alcohol consumption, adjusting their sleeping patterns, and exercising more.

“I used to be a fitness fanatic in my younger days, so I started doing that and like I say I stopped smoking after 40 years and, you know, it was all working, that part of it is very helpful.”

PID47, male, high user

#### Change in attitudes towards medication

Six participants reported being more medically informed as a result of the medication module and subsequently changing their attitudes towards taking medication. Two participants reported being more confident to try medication and more willing to experiment with medication.

“I was very resistant to the idea of medication although I’d sort of reluctantly agreed to it, and it did, I did feel much more confident in the idea of medication, and more willing to, you know, experiment, or try that as a solution.”

PID14, male, high user

#### Facilitation of greater understanding and support from others

Twelve participants chose to share the content of the programme with others, mostly through showing family and friends the website. Many participants who shared the content of the programme with a family member, partner or friend reported that doing so was useful because it facilitated communication, understanding and support. The first 2 modules on diagnosis and aetiology were commonly shared with partners.

*“*[I] *know my triggers, um, such as stress and sleep, exercise, alcohol intake, and so do my family now, and so do my work colleagues which is great. […] They, the ones that I’m very close to in work, can pick up on when I’m perhaps even heading for a low, before, well, not before I do, but at the same time that I can see it, they will point it out to me.”*

PID78, male, high user

## Discussion

### Main findings

We identified several key themes within each domain of enquiry

FEASIBILITY:

Accessibility and flexibility

The effect of illness on engagement with the programme

The importance of accessing the programme in a private environment

ACCEPTABILITY:

Clarity and quality of content

Dislike of actors’ acting

Difficulty with the interactive “life chart” exercise

Lack of activity on the forum

Presentation of lithium within the medication module

Preferences for alternatives to the computer-based format

Internet-based psychoeducation lacks the sociability of group-based learning

Groups of people with mental illness are unappealing

Internet-based psychoeducation may be more acceptable than group-based psychoeducation for those newly diagnosed

IMPACT:

Minimal contribution to existing knowledge for those with a long-standing diagnosis

Potential greater impact for those with a recent diagnosis

Greater knowledge of bipolar disorder

Improved self-awareness

Behaviour change as a result of the programme

Change in attitudes towards medication

Facilitation of greater understanding and support from others

#### Feasibility

The implementation of the programme was found to be feasible for those who had access to a computer and were willing and sufficiently able to use a computer. The programme was commended on its accessibility and ease of use. Some participants specified that they required privacy when accessing the programme and others commented that they needed to be well enough to undertake the programme in order to concentrate on it. This confirms the finding of a recent study examining the predictors of attrition of an online bipolar education programme where the most common theme arising from interviews was that the nature of the illness made it difficult for some participants to continue their involvement with the programme [24].

#### Acceptability

The programme was found to be acceptable to participants who were satisfied overall with the content and presentation of the programme and made suggestions for improvements. The presentation of the programme was reported to be professional and clear, and the pace of the modules and the time between modules was regarded as acceptable. Revisions of the programme should focus on the use of actors, the forum, the life chart exercise, and the content of the medication module. An alternative format of the programme, such as group-based psychoeducation or a psychoeducation manual for patients, should be offered for those who are resistant to using a computer, perhaps especially for older individuals who may not be familiar with using the internet.

We found that many participants who preferred internet-based psychoeducation for bipolar disorder felt that interacting with groups of people with mental illness was not an appealing prospect for them. These individuals did not easily identify with people whom they considered to have a serious mental illness and felt that they would be frightened or easily upset by witnessing others with a more severe form of the disorder. Additionally, we found that some participants regarded online psychoeducation as more suitable than group-based psychoeducation for those newly diagnosed, due to the accessibility, flexibility, privacy, and anonymity of online psychoeducation and the stigma associated with groups of people with mental illness. This finding supports the results of a study of computerised cognitive behavioural therapy for depression, in which freedom and anonymity were found to be motivating factors contributing to adherence to online self-help [25].

Participants who expressed a preference for group-based face-to-face psychoeducation preferred the sociability of group-based learning. Many of these individuals were resistant to using a computer. There were clear limitations within the forum, which was not as effective as we had hoped in providing peer and social support. A key insight from the focus groups which were held initially to develop the content and format of this intervention was that social support via an online forum was desirable [5]. The purpose of the forum was to enable participants to discuss their experiences of the modules and their illness with a view to enhancing their learning experiences and reducing any feelings of social isolation or stigma [5]. It is apparent that the forum did not serve this purpose, perhaps because of its lack of critical mass (only half of all trial participants in the intervention arm contributed to the forum [16]), its lack of input from professionals and because for some it was not viewed as an appropriate medium for social support. It is of interest that previous research in the field of internet-based cancer support interventions has identified that newly diagnosed individuals are much more likely to participate in online discussion groups rather than take part in face-to-face support groups [26-28]. Our online forum may therefore be better suited to those at an early stage of illness.

#### Impact

Participants’ capacity to benefit from the programme was reduced for those who had been diagnosed with bipolar disorder many years previously. For these participants the programme contributed little to their existing knowledge of bipolar disorder. The programme was found to impact to some degree upon some participants’ insight into their illness – specifically, their knowledge of self-management techniques, their self-awareness, and their acceptance of their illness. Furthermore, the programme impacted on health behaviours, lifestyles and personal routines and attitudes towards medication. Many participants chose to share the content of the programme with others, which they reported as having contributed to the quality of their personal relationships through enhanced communication and understanding.

### Strengths and limitations

This study is the first qualitative enquiry to evaluate an online psychoeducation programme for bipolar disorder. Interviews enabled both high and low users of the programme to elaborate on their experiences of it, which gave insights into how the programme was experienced, what was considered to be effective and areas for improvement. Respondents commented on contextual factors which might influence the acceptability and efficacy of the intervention in practice, as well as fidelity of delivery. The main limitation of the study is that the majority of participants recruited for the trial were not recently diagnosed with bipolar disorder and were already familiar with much of the material presented. This affected the extent to which some participants were able to benefit from the programme, and may have affected the outcomes of the trial [16]. Furthermore, the format of the semi-structured interview may have restricted participants’ responses, and participants may have forgotten aspects of the programme in the 6 to 8 months between completion of the intervention and being interviewed. We acknowledge that this paper only provides an overview of the themes elicited from the interviews. Future research will address key themes in significantly greater depth, which will enhance readers’ understandings of the complexities and implications of certain issues.

## Conclusions

An online psychoeducation package for bipolar disorder such as *Beating Bipolar* is feasible and acceptable to patients who are amenable to computerised learning and have access to a computer and may be particularly suited to early intervention. Ideally, alternative formats should also be made available to patients who would prefer either written materials or a group-based, face-to-face learning environment. Future research should evaluate an intervention of this kind specifically targeted at those who have been recently diagnosed with bipolar disorder and explore optimal ways to provide online peer and social support. Overall, internet-based interventions of this kind have considerable potential to deliver high quality self-management and psychoeducational support for mental health problems such as bipolar disorder at relatively low cost.

## Appendix 1

Semi-structured interview schedule

INITIAL QUESTIONS:

How are you doing at the moment?

Have you felt better or worse since April, or do you feel the same as you felt then?

*If participant feels better or worse:* To what extent?

A) ACCESS

Could you access the programme?

To what extent do you feel competent in using a computer?

Did you access the programme at home or in a public venue (such as a library or internet cafÃ©)?

*If participant accessed programme in public venue:* Did you feel that your privacy was compromised as a result of accessing the programme a public venue?

How much of the programme did you do?

(Can you tell me which modules you did?)

(Did you finish the modules?)

(Did you skip any modules?)

*If participant did not continue with programme:* Why did you decide not to continue with the programme?

Did you need assistance from anyone with any aspect(s) of the programme?

*If so:* who; with what; why?

Did you use the forum?

(Did you contribute to the forum or just read it?)

What are your impressions of the forum?

How could the forum be improved?

Do you, or would you, still log in to the website? (*If so:* Why?)

B) REFLECTIONS ON MOOD

Did you experience a significant high or low before, during, or after the programme (such as depression or mania)?

*If so:* Do you feel that this may have impacted on your ability to benefit from the programme?

C) GENERAL

Why did you want to undertake the programme?

What did you like about the programme?

What didn’t you like about the programme?

Were there aspects you found to be particularly helpful?

Were there aspects you found to be frustrating?

Overall, would you say you have benefitted from undertaking the programme?

D) CONTENT

Could you understand the content of the modules? (*Ask for elaboration if necessary*)

Were some modules easier to grasp than others? (*If so:* which were easier and why; which more difficult and why)

Did you have any difficulty paying attention to the modules? (*If so:* why?)

Were any modules more interesting than other modules? (*If so:* why?)

Were any modules more relevant to you than other modules? (*If so:* why; and why were other modules less relevant?)

Have you any other comments or suggestions for improvement regarding the content of the modules?

Did you share the content of any of the modules with anyone? (*If so:* which [aspects of] modules, why, and how?)

Did the programme impact on your relationship with your family?

Since using the programme have you made any lifestyle changes? (*If so:* what are they? *And:* what triggered this?)

E) PRESENTATION FORMAT

What are your impressions of the visual appearance of the programme? *(Probe: videos; tasks to do)*

Was the pace of each module okay, or too fast or too slow?

Did the programme run smoothly on your computer?

Did you find any aspect of the design of the programme particularly engaging? (*If so:* which?)

Did you find any aspect of the design of the programme particularly frustrating? (*If so:* which?)

Have you any other comments or suggestions about the presentation of the modules?

Was the gap between modules about right?

F) INSIGHT

Has the programme as a whole, or any module or modules in particular, impacted upon your understanding of bipolar disorder? (Can you tell me more?)

As a result of the programme are you more aware of how to manage your condition? (Can you tell me more?)

As a result of the programme have you modified aspects of your behaviour or your routine? (Can you tell me more?)

Has your attitude towards medication changed as a result of the programme? (Can you tell me more?)

G) SUGGESTIONS FOR IMPROVEMENT

Have you any other comments or suggestions for improvements?

H) RECOMMENDATIONS

Do you think the programme may help others with bipolar disorder?

Would you recommend the programme to others with bipolar disorder? (*If so:* why?)

In the future should the programme be accessible to people with bipolar disorder via the NHS?

Can you think of characteristics of some participants which may prevent them from fully benefiting from this programme? *(Prompt for elaboration if necessary)*

I) ADDITIONAL SUPPORT

Aside from the programme, since July 2009 has anything or anyone else provided you with additional support to manage your bipolar disorder?

(*If asked, give examples*)

*If so:* How did this help?

If you had been given the choice of either Beating Bipolar the online programme or a group-based programme (where you may have up to 15 people with bipolar disorder learning together under the direction of a clinician) which format would you have preferred?

Why?

## Competing interests

All authors report that they have no competing interests.

## Authors’ contributions

RP recruited participants, conducted the interviews, and coded and analysed the data. RP, DS and SS sought agreement on the design and methods used for the study. RP drafted the manuscript. DS and SS contributed to the final manuscript. All authors read and approved the final manuscript.

## Pre-publication history

The pre-publication history for this paper can be accessed here:

http://www.biomedcentral.com/1471-244X/12/139/prepub
